# Optical System Error Analysis and Calibration Method of High-Accuracy Star Trackers

**DOI:** 10.3390/s130404598

**Published:** 2013-04-08

**Authors:** Ting Sun, Fei Xing, Zheng You

**Affiliations:** 1 Department of Precision Instruments and Mechanology, Tsinghua University, Beijing 100084, China; E-Mails: sunting09@mails.tsinghua.edu.cn (T.S.); yz-dpi@mail.tsinghua.edu.cn (Z.Y.); 2 State Key Laboratory of Precision Measurement Technology and Instruments, Tsinghua University, Beijing 100084, China

**Keywords:** star tracker, error analysis, calibration, parameter estimation

## Abstract

The star tracker is a high-accuracy attitude measurement device widely used in spacecraft. Its performance depends largely on the precision of the optical system parameters. Therefore, the analysis of the optical system parameter errors and a precise calibration model are crucial to the accuracy of the star tracker. Research in this field is relatively lacking a systematic and universal analysis up to now. This paper proposes in detail an approach for the synthetic error analysis of the star tracker, without the complicated theoretical derivation. This approach can determine the error propagation relationship of the star tracker, and can build intuitively and systematically an error model. The analysis results can be used as a foundation and a guide for the optical design, calibration, and compensation of the star tracker. A calibration experiment is designed and conducted. Excellent calibration results are achieved based on the calibration model. To summarize, the error analysis approach and the calibration method are proved to be adequate and precise, and could provide an important guarantee for the design, manufacture, and measurement of high-accuracy star trackers.

## 1. Introduction

With the development of Earth-observing satellites and deep-space exploration satellites, requirements for attitude measurement accuracy are increasing. Thus, error analysis of the accuracy and calibration of the star tracker have become particularly important.

At present, research and analysis of the effect factors on the star tracker accuracy are being conducted. References [1] and [2] provides a general overview of the effects of the optical parameters. References [3] and [4] use a geometric method to establish a complicated error model, and obtain variations in accuracy for a certain range of optical parameters, but most of the existing analysis methods discuss the effects of factors separately and qualitatively. Up to now systemic error analysis and accurate error propagation model are inadequate.

Factors such as misalignment, aberration, instrument aging and temperature effects [5] could cause a departure of the star trackers from the ideal pinhole image model, and contribute to the attitude measurement error. Misalignment and aberration are time-independent, or static errors, which need to be calibrated prior to launch, and can be called ground-based calibration. By contrast, instrument aging and temperature effects are time-varying, or dynamic errors, which must be calibrated in real time, and can be called on-orbit calibration. This paper focuses only on the ground-based calibration method.

The ground-based calibration of star trackers generally includes real night sky observation and laboratory calibration. Real night sky observation can take advantage of the characteristics of the star tracker utilizing the star angular distance. This method is relatively easy to apply, whereas the model parameters interact with one another. Obtaining the global maximum is difficult, and this method is greatly influenced by the environment. Laboratory calibration could use a star simulator as the source. However, it is not easy to manufacture a high-accuracy star simulator.

Camera calibration techniques [6–10] can be choices for calibrating the star tracker considering that they are both optical imaging devices. However, there are problems for the star tracker to apply the calibration methods of the camera. First, most of these methods need to establish a complicated calibration model with scores of parameters. Good calibration results depend largely on the initial values and large amounts of calculation are needed for the optimization. Observability and convergence can be problematic. Second, the star tracker focuses more on the accuracy of the position of the image point, while the camera focuses more on the MTF or other image quality. Since the noteworthy parameters of the calibration methods of the camera and the star tracker are not exactly the same, the accuracy of general camera calibration techniques is not enough for the calibration requirement of the star tracker, which is one of the highest precision attitude measurement devices on the satellite. Moreover, majority of the camera calibration techniques have not considered the inclination of the image plane.

Last but not the least, the optical imaging principle and focus matters of the star tracker and the camera are not the same due to their functions. The camera uses a finite distance imaging mode, while the star tracker adopts an infinite distance imaging mode. General camera calibration methods are not suitable for the star tracker. Taking reference [11] as an example, the cubic 3-D calibration object applies to camera calibration as the camera can take a clear photograph of a finite distance object, but the star tracker is used to take pictures of infinite distance stars, so it cannot take a clear photograph of the 3-D calibration object. Even though there are a few reports about how to add another high accuracy lens to make this finite imaging calibration method apply to the star tracker, the accuracy and the position of the added lens, the accuracy of the 3-D cubic object all need to be discussed. These bring new troubles and are not easy to carry out.

Therefore, the calibration method provided in literature [11] or other similar camera calibration methods work better on short focal length, small view field camera. Convenient calibration methods for large FOV and high accuracy star tracker are still problems needed to be figure out. The calibration method using composite mode of high accuracy autocollimator theodolite and the features of the star tracker proposed in the manuscript is a good choice for this topic.

To summarize, the literature on the analysis and evaluation of the error sources of star trackers has not been adequate until now. This paper proposes a systematic method for weight analysis of the error source. Optical parameters that play key roles in the accuracy of the star tracker (such as the principal point deviation, focal length error, imaging plane inclination error and distortion) [1,12] are analyzed by the proposed method. From the analysis results, a calibration method is put forward. The calibration can separate the radial distortion from the image plane inclination, thus the optimization processes are simplified. The calibration result proves that the analysis of the optical systematic error and the calibration method for the high-accuracy star trackers proposed in this paper are reasonable and adequate, and can improve the accuracy of the star tracker.

## 2. Star Tracker Mesurement Model

The star tracker is a high-accuracy attitude measurement device, which considers the stars as the measuring object. It obtains the direction vector from the celestial inertial coordinate system by detecting the different locations of the stars on the celestial sphere. After many years of astronomical observations, star positions on the celestial sphere are predictable. Stars in the celestial sphere coordinate system can be expressed in the right ascension and declination (*α*,*δ*). Based on the relationship between the rectangular coordinate system and the spherical coordinate system, the direction vector of the stars in the rectangular coordinate system is expressed as follows:

(1) $$v=\left[\begin{array}{c} \cos\alpha \cos\delta \\ \sin\alpha \cos\delta \\ \sin\delta \end{array}\right]$$

Navigation stars are selected from the star catalog to meet the imaging requirement, and their data are stored in the memory of the star tracker.

**Figure 1.**
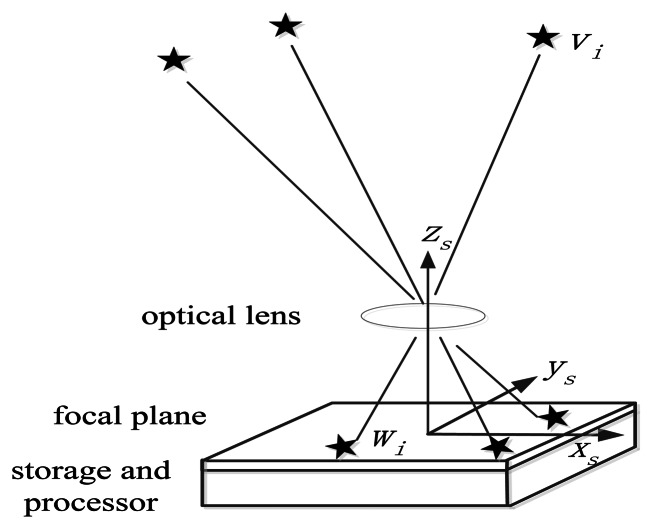
Star tracker ideal imaging model.

When a star tracker with attitude matrix *A* is in the celestial coordinate system, the ideal measurement model of the star tracker can be considered as a pinhole imaging system. Navigation star *S_i_* with direction vector *v_i_* under the celestial coordinate system can be detected through the lens, whereas the vector of its image can be expressed as *w_i_* in the star tracker coordinate system, as shown in Figure 1.

The position of the principal point of the star tracker on the image plane is (*x*_0_, *y*_0_). The position of the image point of navigation star *s_i_* on the image plane is (*x_i_*, *y_i_*). The focal length of the star tracker is *f*. Vector *w_i_* can be expressed as follows [13]:

(2) $$w_{i}=\frac{1}{\sqrt{{\left(x_{i}-x_{0}\right)}^{2}+{\left(y_{i}-y_{0}\right)}^{2}+f^{2}}}\left[\begin{array}{c} -(x_{i}-x_{0})\\ -(y_{i}-y_{0})\\ f \end{array}\right]$$

The relationship between *w_i_* and *v_i_* under the ideal condition can be expressed as follows, where *A* is the attitude matrix of the star tracker:

(3) $$w_{i}=Av_{i}$$

When the number of navigation stars is more than two, the attitude matrix can be solved by the QUEST algorithm [14]. In this method, the optimal attitude matrix *A_q_* in the inertial space of the star tracker can be calculated.

## 3. Star Tracker Error Analysis

### 3.1. Summary of the Error Sources of the Star Tracker

The existence of errors and noise in the system are inevitable. According to the pinhole model shown in Figure 1 and Equation (2), the factors that directly affect the results of the attitude measurement of the star tracker include the extraction error of star point position, principal point error, error of focal length, direction vectors of the navigation stars, and attitude solution algorithm error. The accuracy is also related to the number of stars in the field of view (FOV). Further, the effect factors of the star tracker are classified as follows [1–5]:

#### 3.1.1. Star Vector Measurement Error

Star vector measurement error concerns the accuracy of vector *w_i_* in Equation (3). Star vector measurement error includes:

(1) Extraction error of the star point position
  The process in which the star tracker detects the navigation stars includes background radiation, optical systems, photoelectric detectors and signal processing. Each segment affects the extraction quality of the target signal.
  Stars are far from the Earth, thus, starlight rays are considered as parallel light rays and can converge to a point on the focal plane. However, most star trackers adopt the defocus form [15] so that the image point can be diffused to cover several pixels. Using the signal energy of multiple pixels enables the star point-extraction accuracy to achieve a sub-pixel level. This process means that after considering the various factors, the extraction error of the star tracker can be considered as 0.1 pixels [16]. This concept lays the foundation of the following analysis.
(2) Star tracker optical parameter errors
  The star tracker system cannot achieve the ideal image model because of the principal point deviation, focal length error, inclination of the image plane, distortion in practical use. Therefore, it is necessary to establish a calibration model for above parameter errors, and analyze parameter error and model error.

#### 3.1.2. Star Catalog Error

Star catalog errors concern the accuracy of vector *v_i_* in Equation (3). The number of stars is very large; hence, we must select the appropriate ones for storage in the memory and meet the performance requirements of the star tracker. The different stars selected may influence the star numbers that appear in the FOV. The star catalog set-up time could also contribute small errors in the direction vectors of the stars in the celestial coordinate system. But the influence can be ignored if the star catalog can be corrected every once in a while considering proper motion of stars.

#### 3.1.3. Star Tracker Internal Algorithm Error

Star tracker internal algorithm error concerns the accuracy of the final attitude matrix *A* of the star tracker. However, algorithm errors such as star pattern recognition methods and attitude solution algorithm are irrelevant to this work. Among the errors enumerated above, those described in Section 3.1.1 exert a relatively larger effect. The error analysis in this paper focuses mainly on this error source.

### 3.2. Error Propagation Model

In the following analysis, we use the angle measurement error (*ξ_A_*) to represent the star tracker accuracy. According to the pinhole image model, *ξ_A_* is expressed as follows: the change in the angle between the incident ray and the optical axis in the FOV is called the angle of real light change (*ξ_AR_*), and the light calculated from the star position and focal length is called the calculated light. The change in the angle between the calculated light and the optical axis is called the angle of calculated light change (*ξ_AC_*). The deviation in the *ξ_AR_* and *ξ_AC_* is called the *ξ_A_*. The *ξ_A_* represents the star tracker attitude measurement accuracy. The optical parameter errors such as the principal point error, error of focal length, inclination of the image plane and distortion influence the *ξ_A_* and cause a difference betwe en the *ξ_AR_* and *ξ_AC_*. Therefore, analysis of the effect of the different optical parameter errors on *ξ_A_* could identify the key factors that must be calibrated, which is important in improving the star tracker accuracy and can provide optimization guidelines for the star tracker design.

**Figure 2.**
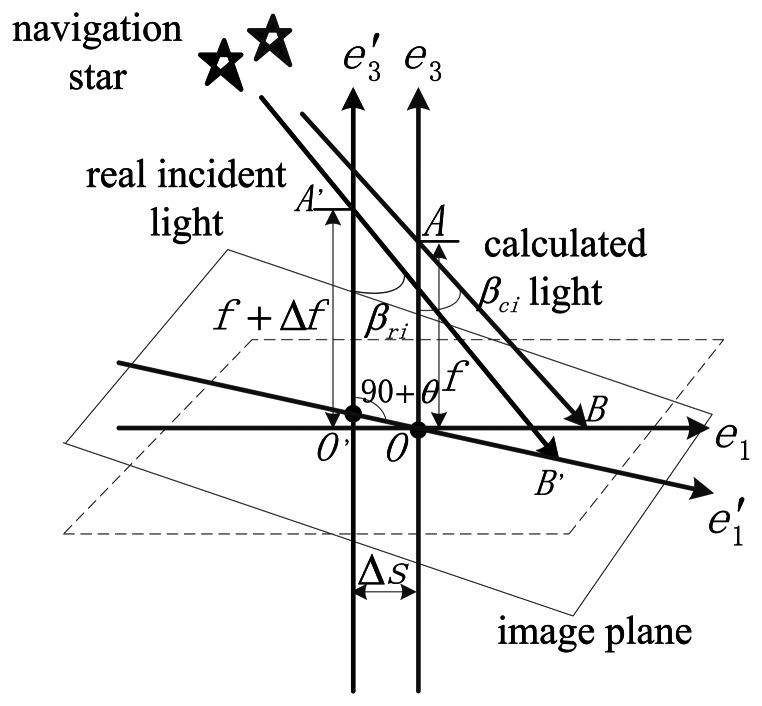
Sketch of complete error propagation.

Figure 2 shows the sketch of complete error propagation. *β_ri_* is defined as the angle between the incident ray and the optical axis. The initial value of *β_ri_* is *β_r0_*, *β_r0_* =0°. The maximum value of *β_ri_* is equal to the angle of the FOV. Δ*β_ri_* is the *ξ_AR_*, and Δ*β_ri_* = *β_ri_* − *β_r0_*. *β_ci_* is the angle between the calculated light and the optical axis. Δ*β_ci_* is the *ξ_AC_*, and Δ*β_ci_* = *β_ci_* − *β_c0_*. We made the following assumptions: axis *e_1_* is along the direction of the maximum error of the principal point, and the inclination angle of the image plane in this direction is also the maximum of all directions. This can represent the worst case of error conditions. Axis *e_3_* is the ideal optical axis of the system, and *e^′^_3_* represents the actual optical axis. Δ*s* represents the deviation of the optical axis. Δ*f* is the deviation of the focal length. Δ*x* represents the star point-extraction error and Δ*d* is the distortion value. The distortion discussed in this paper concerns radial distortion only.

(4) $$O\prime B\prime =\frac{f+\Delta f+\Delta s\cdot \tan(\theta )}{\sin(90-\theta -\beta_{ri})}\cdot \sin(\beta_{ri})+\Delta x+\Delta d$$

(5) OB=OB′=O′B′−O′O

Angle *β_ci_* is obtained as:

(6) $$\beta_{ci}=\arctan\left(\frac{OB}{f}\right)$$

The *ξ_A_* is calculated based on the above analysis:

(7) $$\xi_{A}=\arctan\left(\frac{\left(\frac{f+\Delta f+\Delta s\cdot \tan(\theta )}{\cos(\theta +\beta_{ri})}\cdot \sin(\beta_{ri})+\frac{\Delta s}{\cos(\theta )}+\Delta x+\Delta d\right)}{f}\right)-\arctan\left(\frac{\Delta s}{f\;\cos(\theta )}\right)-\beta_{ri}$$

When the actual optical axis is along the positive direction of the ideal optical axis, Δ*s* is defined as positive. When the inclination angle *θ* is in the clockwise direction, it is defined as positive. Another *ξ_A_* form is expressed as follows:

(8) $$\xi_{P}=O\prime B\prime -f\;\tan(\beta_{ri})=\frac{f+\Delta f+\Delta s\cdot \tan(\theta )}{\cos(\theta +\beta_{ri})}\cdot \sin(\beta_{ri})+\Delta x+\Delta d-f\;\tan(\beta_{ri})$$

We define *ξ_P_* as the position measurement error of the incident light ray. *ξ_A_* and *ξ_P_* describe the measurement error in different views, however, they can be transformed from one form to the other. *ξ_A_* is more concerned with the accuracy of the star tracker, whereas *ξ_P_* is more suitable for using in the calibration.

### 3.3. Star Tracker Optical Parameter Errors Simulation

We adopt two methods to discuss the error effects. First, we use the Monte Carlo (MC) stochastic modeling method. In this method, it is assumed that the errors after calibration, such as noise, inclination angle, focal length and principal point errors are random errors. These errors are considered to coincide with the normal distribution. In addition, the number of stars that can be captured in the sky is more than 6,000. Therefore, it is reasonable to consider the incident angle in the FOV as uniformly distributed. Based on the two statistical assumptions, we can combine the geometry and MC random models, and develop the complete error effect analysis. Second, we use the maximum error method to prove the simulation result of the MC method. This method can easily identify the error distribution of the different incident angles. Analysis of the position of the maximum error can also provide information for further study. The object analyzed in this paper is a star tracker of 7″ accuracy and the FOV is 17°. The focal length of the system is approximately 49.74 mm. The star tracker adopts the APS CMOS image sensor with 1,024 × 1,024 pixels, and the size of each pixel is 0.015 mm.

#### 3.3.1. MC Error Analysis Method

The MC method [17] used the statistical rule of random numbers for the calculation and simulation. The following subjects analyze the single factor and the combination of factors using the MC method. Using a 1,000,000 times simulation, the statistical results are obtained. The simulation is conducted with an 8.5° incident angle.

Then, combination error analysis using the MC stochastic simulation is conducted. The *ξ_A_* satisfies the distribution rule: *μ* = −0.0225, *σ* = 4.4218″. The accuracy is in the range of *μ*−3*σ*∼*μ*+3*σ* = −13.2881∼13.2430″. Considering that at least 4 stars can be captured in the normal working state, the simulation boresight accuracy can be calculated as: $-13.2881/\sqrt{4}∼13.2430/\sqrt{4}=(-6.6440\prime \prime ∼6.6215\prime \prime )$. Thus, the allocation of permissible errors of the star tracker with a 7″ accuracy is obtained.

**Figure 3.**
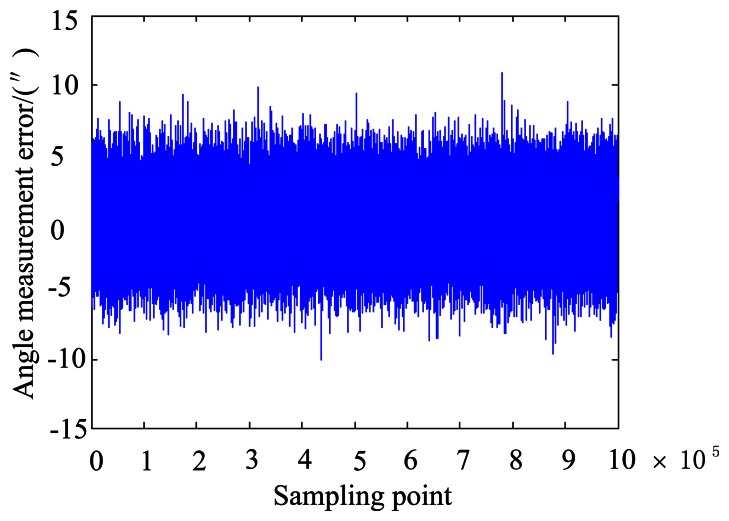
Synthetic analysis result using MC stochastic simulation.

Table 1 and Figure 3 show that, for the analyzed system, error of star point extraction, error of principal point, error of focal length, error of inclination of the image plane and distortion are respectively distributed in the range of 0.1 pixels, 4.5 pixels, 0.6 pixels, 0.075° and twenty thousandth, they bring the *ξ_A_* to the same level. Considering their combined effects can ensure the accuracy of the star tracker.

#### 3.3.2. Simulation of the Maximum Error Method

**Figure 4.**
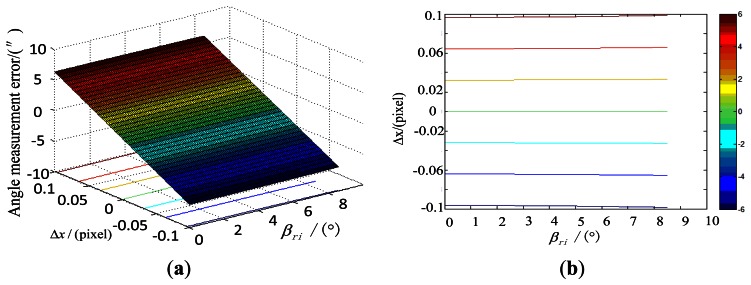
Influence of the star point extraction error on the star tracker accuracy. (**a**) is obtained when the star point extraction error Δ*x* is among the range from −0.1 pixels to 0.1 pixels; (**b**) is the contour line of (a).

We conduct a simulation using Maximum Error Method to compare with the method in Section 3.3.1 based on the error propagation model in Section 3.2 and the system parameters of the star tracker, as well as the range of the star point extraction error, the principal point error, focal length error, inclination of the image plane and distortion. The incident angle *β_ri_* is in the range of 0–8.5° and the focal length is approximately 49.74 mm. Under the simulation conditions, the effect of the error of star point extraction on the star tracker accuracy, along with the incident angle is shown in Figure 4.

**Figure 5.**
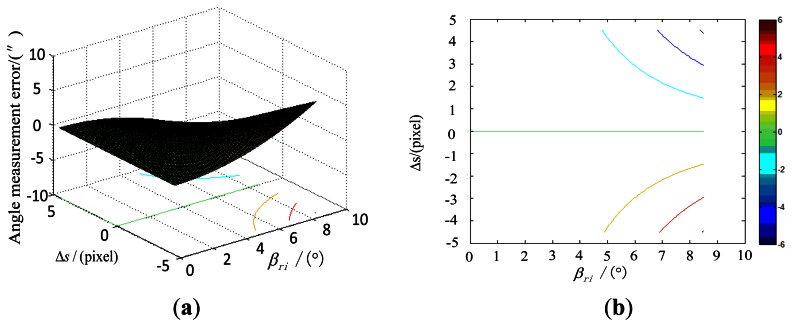
Influence of the principal point error on the star tracker accuracy. (**a**) is obtained when Δs is among the range from −4.5pixels to 4.5 pixels; (**b**) is the contour line of (a).

Under the simulation conditions, the effect of the error of the principal point on the star tracker accuracy, along with the incident angle is shown in Figure 5.

**Figure 6.**
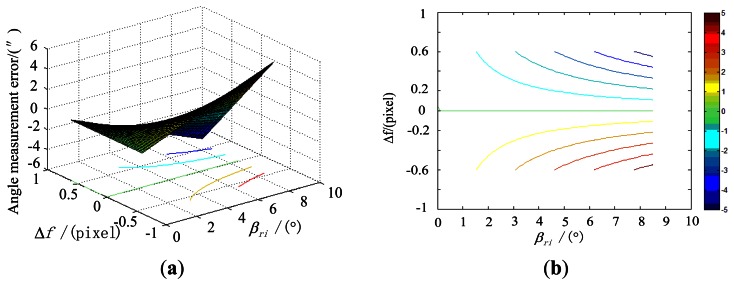
Influence of the focal length error on the star tracker accuracy. (**a**) is obtained when Δf is within the range from −0.6 pixels to 0.6 pixels; (**b**) is the contour line of (a).

Under the simulation conditions, the effect of the error of focal length on the star tracker accuracy, along with the incident angle is shown in Figure 6.

**Figure 7.**
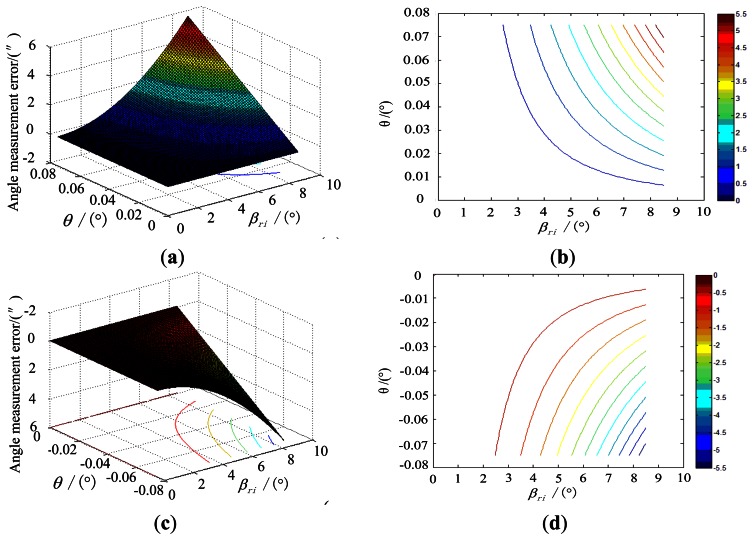
Influence of the inclination angle error on the star tracker accuracy. (**a**) is obtained when *θ* is in the range from 0° to 0.075°.(**b**) is the contour line of (a). (**c**) is obtained when *θ* is in the range from −0.075° to 0°, and (**d**) is the contour line of (c).

Under the simulation conditions, the effect of the error of inclination image plane on the star tracker accuracy, along with the incident angle is shown in Figure 7.

**Figure 8.**
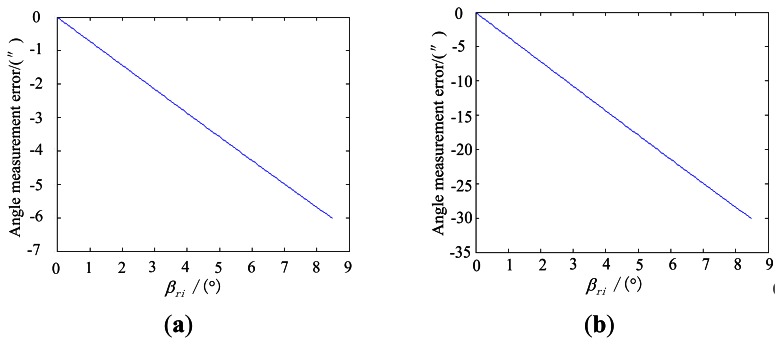
Influence of the distortion on the star sensor accuracy. (**a**) is obtained when the relative distortion is approximately 2/10,000th; and (**b**) is obtained when the relative distortion is approximately 1/1,000th.

Under the simulation conditions, the effect of the error due to distortion on the star tracker accuracy, along with the incident angle is shown in Figure 8.

The simulation results show that the error effects obtained by the maximum error method agree with the results obtained by the MC simulation method. We can also find that inclination of the image plane and the distortion are two key factors that need to be calibrated. The calibration method will be elaborated in the next section.

### 3.4. Conclusion of the Star Tracker Error Analysis

Optical systematic error analysis method proposed in this paper can perform analysis on the sensitivity of factors (such as the error of star position extraction error, position error of principal point, error of focal length, inclination of the image plane and the distortion) that may influence the accuracy of the star tracker.

The above analysis of the system error can be applied in the following areas: (1) reference for the calibration target, *i.e.*, if the five indicators are simultaneously satisfied, the calibration results could meet the requirements; (2) analysis for the highest accuracy of the system; and (3) determining the major factors that emphasized the calibration experiment.

For application (1) above, because of the limitations in the calibration method, the effect factors cannot be separated from one another. Thus, determining whether the five indicators are all satisfied is difficult. Therefore, the proposed method is used primarily in the determination and demonstration of the design indicators. For application (3), some of the restrictions in certain error range can easily meet, such as the principal point position error, whereas satisfying the others are more difficult. These error factors need to be calibrated elaborately, such as the inclination angle of the image plane and the distortion. Therefore, emphasis on the calibration method is related to the system parameters. The calibration method and the processes are designed according to the characteristics of the system, so that the *ξ_A_* or *ξ_P_* of the star tracker is within the design range.

## 4. Laboratory Calibration Method

### 4.1. Star Tracker Calibration Device

The calibration object of this paper is a star tracker with 7″ accuracy. Based on the result of the above analysis, optical parameters of the system are calibrated. The laboratory calibration of the star tracker can be performed using a three-axis turntable and a collimator or an autocollimator theodolite. In essence, their operating principle is the same. However, because the collimator does not have a self-collimation function, which could introduce trouble to the calibration of the principal point, we adopt the autocollimator theodolite.

**Figure 9.**
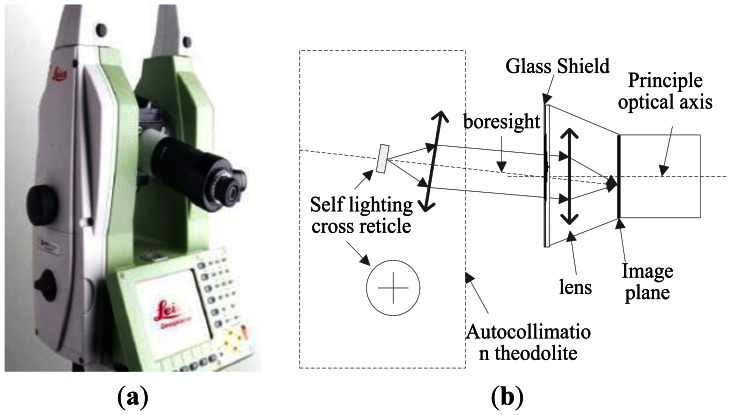
(**a**) External view; (**b**) Internal structure of the autocollimator Theodolite 6100A.

The autocollimator theodolite we employ in the experiment is the Leica 6100A. Figure 9(a) shows its external view. It has a small size, high accuracy 0.5″ and simple operation. We can use its auto-collimation eyepiece to determine whether the crosslines coincide, as shown in Figure 9(b). Other experiment devices consist of the optical table and auxiliary fixtures. It is worth noting that the aperture of the autocollimator should be comparable or larger than the aperture of the star tracker in order to avoid vignetting.

### 4.2. Calibration Algorithm and Experiment

**Figure 10.**
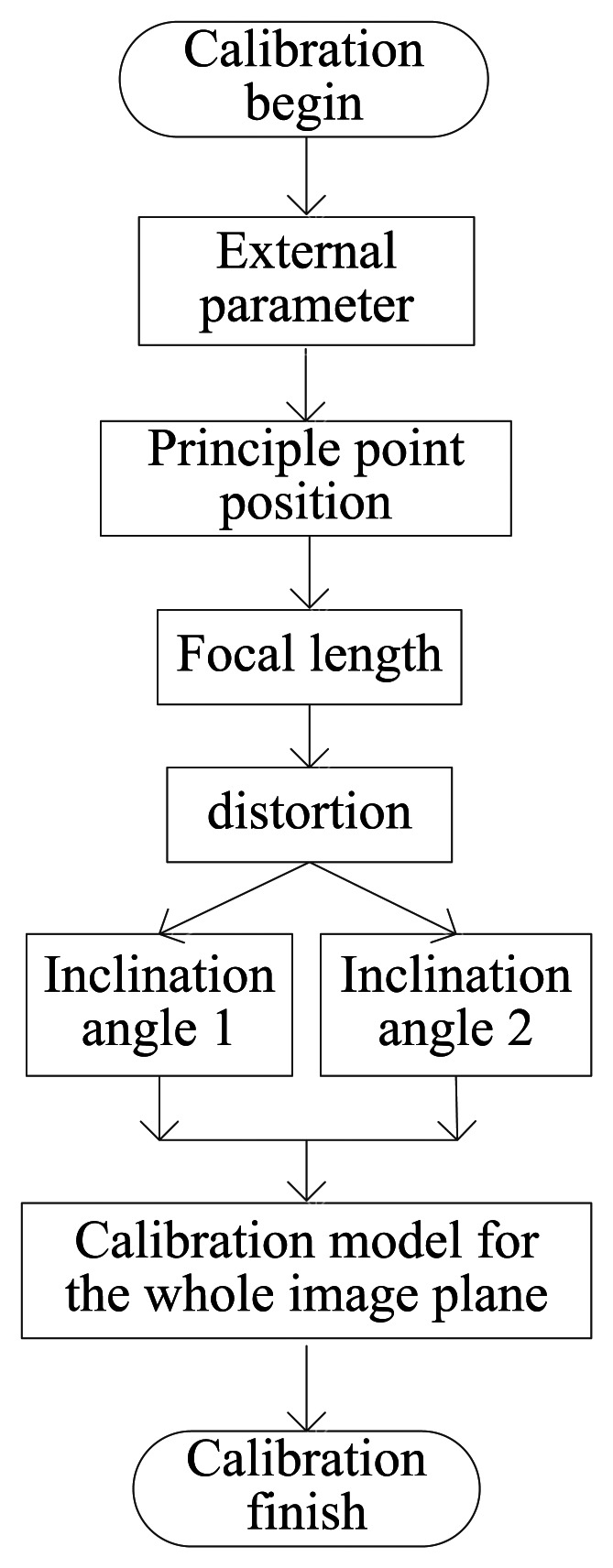
Calibration flow diagram.

According to the analysis in Section 3, the calibration objective should be focused on the inclination of the image plane and the distortion. The basic block diagram of the calibration process is shown in Figure 10.

#### 4.2.1. Coordinate System

**Figure 11.**
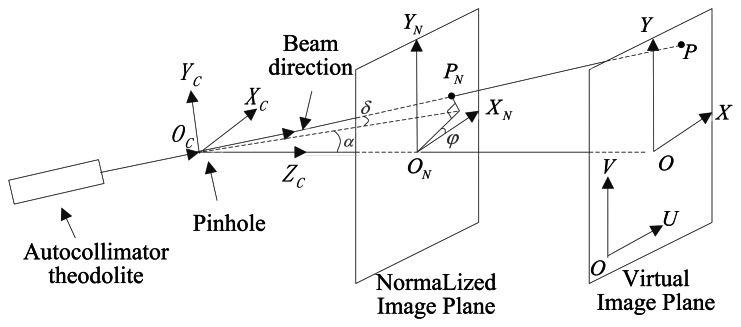
Coordinate systems in the ideal pinhole imaging model.

For better representation of the location and the relationship, we create the coordinate systems as in Figure 11. Axes *X_C_ Y_C_* in coordinate system *CS_C_* represent the movement directions of the outgoing light rays of the theodolite in the two orthogonal axes, respectively. *Z_C_*, *X_C_* and *Y_C_* comply with the right-hand rule. Coordinate system *CS_N_* represents the normalized coordinate of the focal plane. The positive directions of *X_C_ Y_C_* are consistent with the directions of the increase in the image pixel value. The coordinate origin is the same as the optical system principal point. The distance between *CS_N_* and the pinhole is normalized to one. Coordinate system *CS_OXY_* has the same direction as that of *CS_N_*, and the only difference is that the distance between *CS_OXY_* and the pinhole is equal to the focal length *f*. *CS_OUV_* represents the actual coordinate system of the image plane. The positive directions of *u*, *v* are consistent with the directions of the increase in the image pixel value. The coordinate origin is at the zero pixel of the image plane, and the optical system principal point can be expressed as (*u*_0_, *v*_0_). The distance between *CS_OUV_* and the pinhole is *f*. The axes of *CS_O′U′V′_* and *CS_OUV_* have the same direction, with the coordinate origin *O*′ is the same as the principal point. Ideally, *CS_O′U′V′_* coincides with *CS_OXY_*. Angle *α* is the longitude value of the outgoing light ray of theodolite in coordinate system *CS_C_*, whereas *δ* is the latitude value. Angle *φ* is the angle between the axis *X_C_* and axis *X_N_*. Thus, the coordinates of the ideal pinhole imaging model are established as shown in Figure 11. The relationship of the parameters are presented in Equations (9)–(11).

(9) $$\{\begin{array}{c} x_{N}=\tan(\alpha )\cos(\varphi )-\frac{\tan(\delta )}{\cos(\alpha )}\cdot \sin(\varphi )\\ y_{N}=\tan(\alpha )\sin(\varphi )+\frac{\tan(\delta )}{\cos(\alpha )}\cdot \cos(\varphi ) \end{array}$$

(10) $$\{\begin{array}{c} x=fx_{N}=f\cdot (\tan(\alpha )\cos(\varphi )-\frac{\tan(\delta )}{\cos(\alpha )}\cdot \sin(\varphi ))\\ y=fy_{N}=f\cdot (\tan(\alpha )\sin(\varphi )+\frac{\tan(\delta )}{\cos(\alpha )}\cdot \cos(\varphi ) \end{array}$$

(11) $$\{\begin{array}{c} u=x+u_{0}\\ v=y+v_{0} \end{array}$$

**Figure 12.**
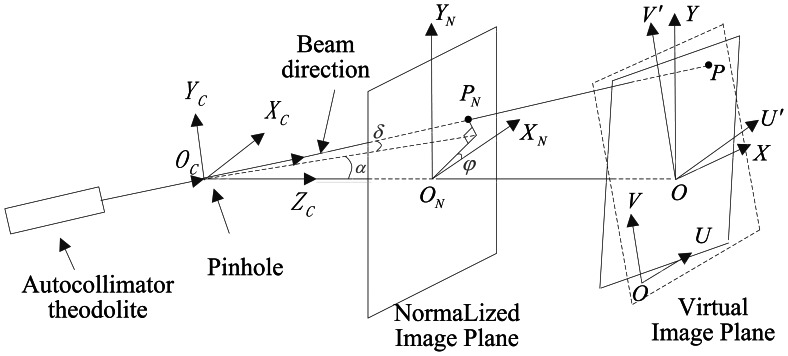
Coordinate systems in the case of errors.

Angle *α* is positive along the positive direction of *X_C_*, whereas it is negative along the negative direction of *X_C_*. Angle *δ* is positive along the positive direction of *Y_C_*, whereas it is negative along the negative direction of *Y_C_*. When the angle from *X_N_* to *X_C_* is counterclockwise, angle *φ* is considered as positive. The ideal pinhole imaging model cannot be achieved in practical application. There are position errors of principal point, focal length error, inclination of image plane and distortion to cause the *CS_O′U′V′_* departure from *CS_OXY_* as shown in Figure 12, and the equations above are invalid. It is necessary to estimate the parameters by calibration.

#### 4.2.2. Description of the Calibration Experiment Operating

First, we adjust the theodolite to ensure that its outgoing light travels only along the longitude direction. The latitude value does not change during this process. Imaging conducted at every 0.5° can yield a series of measured values that determines the external parameters.

**Figure 13.**
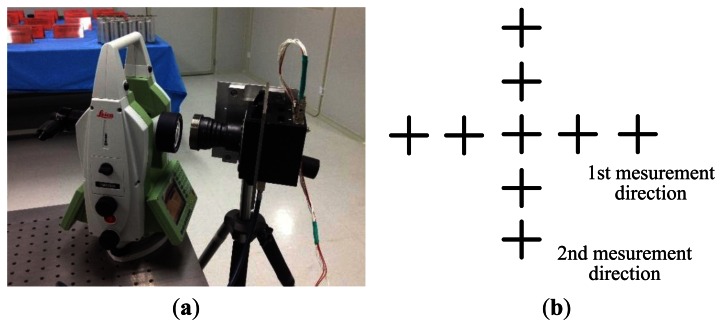
(**a**) Calibration experiment device; (**b**) Imaging method sketch map.

Then, according to the 17° FOV of the star tracker, we adjust the theodolite so that its light travels along the two orthogonal directions as shown in Figure 13 while conducting imaging at every 0.5°. If any of the two directions satisfies the requirement of the first step, two steps can be combined to determine the internal parameter of the star tracker. Because the outgoing rays of the theodolite are crosslines, the images on the image plane appear similar to that shown in Figure 13(b). It is worth mentioning that the star tracker is supposed to be settled on the same optical table with the auto-collimation theodolite to avoid relative vibration. Considering that our experiment is conducted at the State Key Laboratory of Precision Measurement Technology and Instruments, the floor of the laboratory has been treated with vibration isolation. So the relative vibration could be reduced and ignored in the calibration form in Figure 13(a), and it is easier to adjust the relative position of the theodolite and the star tracker with the tripod.

#### 4.2.3. Image Processing

**Figure 14.**
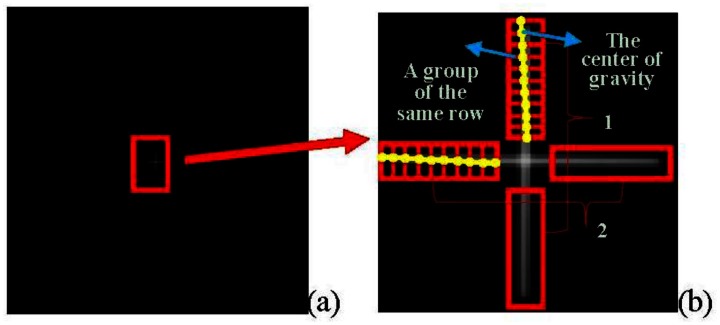
Image of the emergent crossline of the theodolite. (**a**) is original image; (**b**) is partially enlarged view.

**Figure 15.**
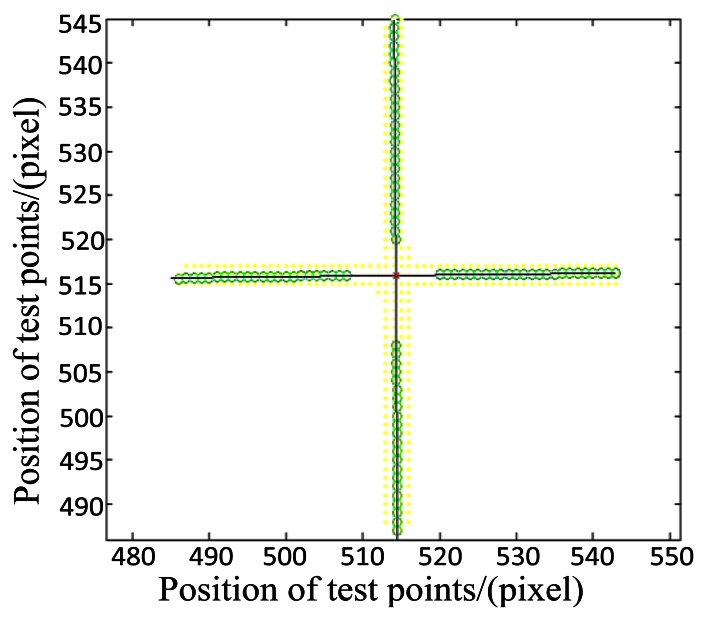
Sketch map of the solution of the gravity center of the cross-line.

The image obtained by the star tracker is shown in Figure 14. An appropriate image processing method should be adopted to obtain the precise center position of the crossline which represents the outgoing ray of the theodolite. For the pixels in the first area, we regard the pixels in the same row as a group, and determine their gray value center of gravity (*i.e.*, weighted average). For the pixels in the second area, we consider pixels in the same column as a group, and also determine their gray value center. The centers of gravity are marked with a circle as shown in Figure 15. Finally, we use the least square method to fit the two straight lines. The point of intersection of the two lines is considered as the center of the crossline. This work provides a basis for further algorithm. Since the light intensity of the theodolite could be adjusted by a knob, obtaining image before experiment and observing whether the image is saturation is also important.

#### 4.2.4. Calibration Process

##### External Parameter Estimation

The series of point coordinate values obtained in Section 4.2.2 can be used to solve angle *φ* between the *X_C_*-axis of coordinate system *CS_C_* and the *X_N_*-axis of *CS_N_*. Symbol *n* represents the number of sampling points:

(12) $$\varphi =\sum_{i=1}^{n}\left(\frac{a\cos(\frac{\left|u_{Ri}-u_{0}\right|}{{\left({\left(u_{Ri}-u_{0}\right)}^{2}+(v_{Ri}-v_{0})\right)}^{1/2}})}{n}\right)$$

Linear fitting can also be adopted to solve the value of *φ* besides Equation (12).

##### Optical Parameter Estimation

**Figure 16.**
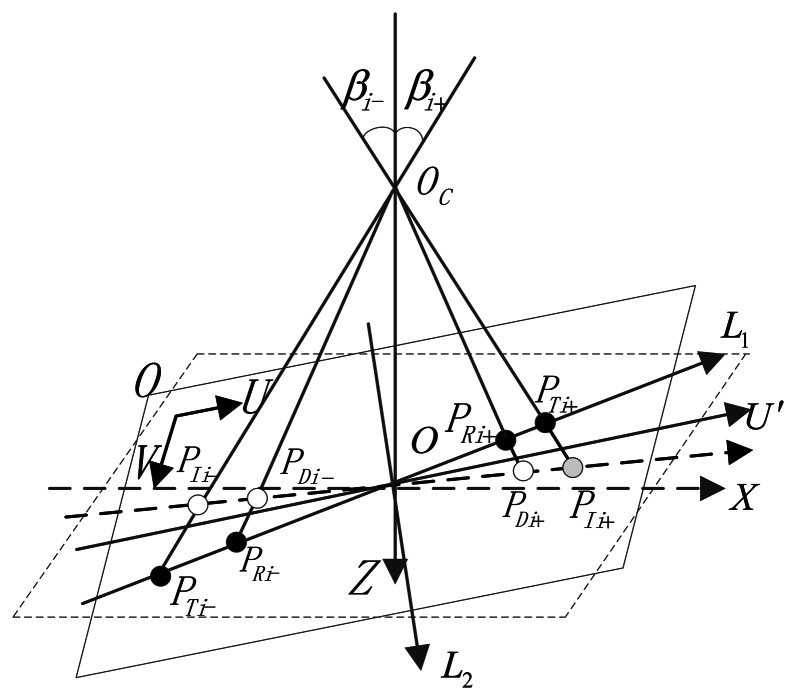
Calibration schematic diagram.

The positions of the series of specific points are obtained according to the above discussion and preparation. The characteristics of these points include the following: the test points are distributed in two orthogonal directions and the test points in the same direction are almost centrosymmetrical. Taking advantage of relationship between the symmetric points, we build a calibration model, shown in Figure 16. In the actual calibration process, we try to make one of *α, δ* equal to zero for simplicity of the calculation. In reality, no restriction is imposed on the theodolite longitude and latitude angle, and the two orthogonal directions formed by the test points do not necessarily coincide with any of the defined coordinates. For the general situation, we name the two orthogonal directions distributed with the test points as *L*_1_ and *L*_2_. *β_i_* is the synthesis angle of the theodolite longitude and latitude. When there are distortion and inclination of the image plane in the system, the imaging model is shown in Figure 16. *β_i_*_+_ and *β_i_*_−_ respectively represent the incident angles of the two centrosymmetric points in the same test direction. *P_Ii_*_+_ and *P_Ii_*_−_ are the ideal image point positions corresponding to the incident light rays when no distortion and inclination of image plane occurs. *P_Di_*_+_ and *P_Di_*_−_ are the positions of the image points when distortion is present but with no inclination of the image plane. *P_Ti_*_+_ and *P_Ti_*_−_ are the positions of the image points with the presence of image plane inclination but with no distortion. *P_Ri_*_+_ and *P_Ri_*_−_ are the positions of the image points when both distortion and inclination of the image plane are present.

**Figure 17.**
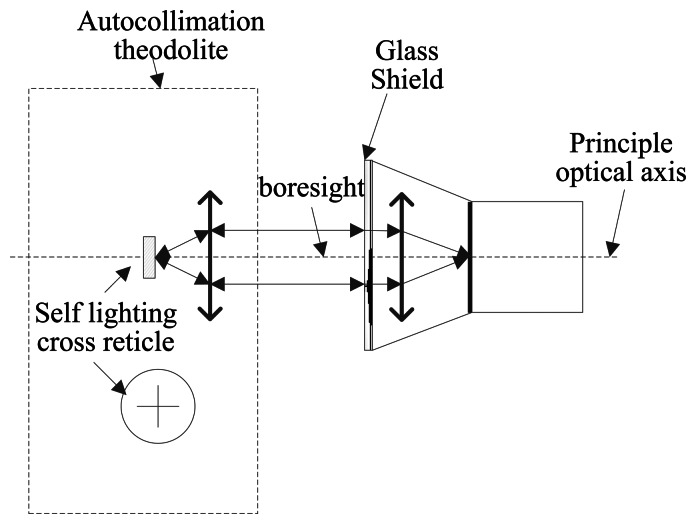
Principal point measurement principle.

**Figure 18.**
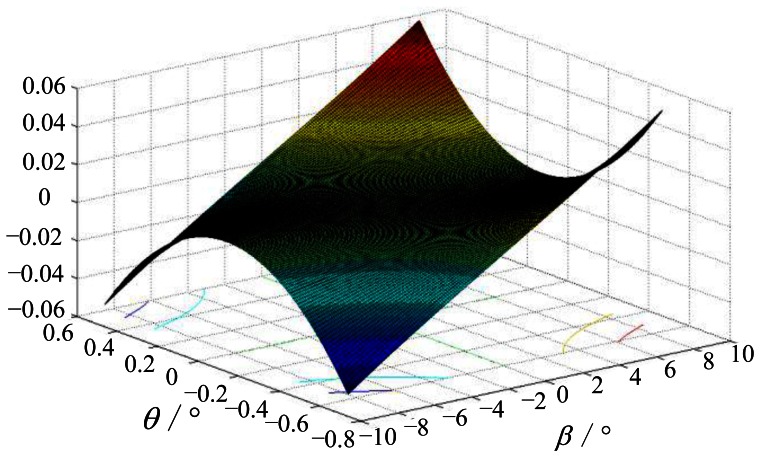
Error between the ideal and real distances of the symmetric points. The *Z*-axis represents the value (|*P_Ti_*_+_*P_Ti_*_−_| − |*P_Ii_*_+_*P_Ii_*_−_|)/pixel.

**Figure 19.**
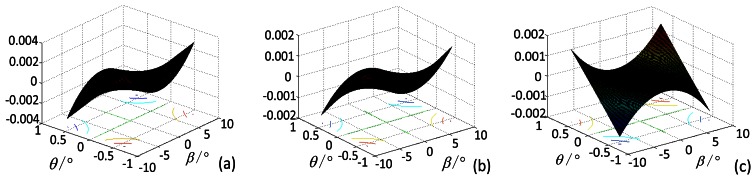
Relationship of the different distortion expressions. *Z*-axis of (**a**) represents (|*P_Ti_*_−_*P_Ri_*_−_| − |*P_Ti_*_+_*P_Ri_*_+_|)/pixel; *Z*-axis of (**b**) represents (|*P_Ti_*_−_*P_Ri_*_−_| − |*P_Ii_*_−_*P_Di_*_−_|)/pixel; *Z*-axis of (**c**) represents (|*P_Ti_*_+_*P_Ri_*_+_| − |*P_Ii_*_−_*P_Di_*_−_|)/pixel.

The longitude and latitude values (*α_i_*, *δ_i_*) corresponding to the incident rays and the coordinate positions (*u_Ri_*, *v_Ri_*) on the image plane can be used to solve the principal point position (*u*_0_, *v*_0_), focal length *f*, distortion coefficients *α*_1_, *α*_2_, *α*_3_, *α*_4_, *α*_5_ and the inclination angle corresponding to the test direction. Finally, the calibration model for the entire image plane is obtained. This method is elaborated as follows:

(1) Firstly, we adjust the auto-collimation theodolite, and ensure that the crossline in the theodolite eyepiece is coincident with the specular reflection image of the star tracker glass shield, as shown in Figure 17. We consider that the boresight of the theodolite is coincident with the spindle of the star tracker lens. The imaging position of the crossline on the image plane is considered as the principal point (*u*_0_, *v*_0_) of the system.
(2) Secondly, we can obtain a series of focal lengths utilizing the incident light in different directions and their image point (*u_Ri_*, *v_Ri_*). The average focal length is considered as the focal length value of the system. *n* represents the number of test points except for the principal point:
  (13) $$\beta_{i}=a\cos(\cos(\alpha_{i})\cos(\delta_{i}))$$
  (14) $$f=\frac{\sum_{i=1}^{n}\frac{{\left({\left(u_{Ri}-u_{0}\right)}^{2}+{\left(v_{Ri}-v_{0}\right)}^{2}\right)}^{1/2}}{\tan(\beta_{i})}}{n}$$
(3) The simulation results shown in Figure 18 prove that when the incident angle |*β_i_*| < 8.5°, inclination angle |θ| < 0.8°, there are following rules of example points: |*P_Ti_*_+_*P_Ti_*_−_| = |*P_Ii_*_+_*P_Ii_*_−_|. As shown in Figure 18, the errors between the two segments are less than 0.1 pixels, which is less than the extraction accuracy of the star tracker. Using the same simulation method, we find that the segments |*P_Ri_*_−_*P_Ti_*_−_|, |*P_Ri_*_+_*P_Ti_*_+_|, |*P_Di_*_+_*P_Ii_*_+_| and |*P_Di_*_−_*P_Ii_*_−_| are equal to one another, whereas the errors are extremely small as shown in Figure 19. Therefore, we consider (|*P_Ii_*_+_*P_Ii_*_−_| − |*P_Ri_*_+_*P_Ri_*_−_|)/2 as the radial distortion value at point *P_Ri_*_+_ or *P_Ri_*_−_, and this concept is an important basis of our calibration method.
  From the multiple groups of symmetrical points, we can obtain optimized distortion coefficients *a*_1_, *a*_2_, *a*_3_, *a*_4_, *a*_5_ by linear least-squares fitting. The form of distortion can be chosen according to the distortion values in different cases. Here we adopt $\sum_{k=1}^{5}a_{k}{\text{R}}_{i}^{k}$ :
  (15) $$R_{i}=\frac{\left|P_{Ri+}P_{Ri-}\right|}{2}$$
  (16) $$\Delta_{i}=\left|P_{Di+}p_{Ii+}\right|=\left|P_{Di-}p_{Ii-}\right|=\left|P_{Ri+}P_{Ti+}\right|=\left|P_{Ri-}P_{Ti-}\right|=\frac{\left(\left|P_{Ii+}P_{Ii-}\right|-\left|P_{Ri+}P_{Ri-}\right|\right)}{2}$$
  (17) $$\{\begin{array}{l} R_{i}=\frac{{\left({\left(u_{Ri+}-u_{Ri-}\right)}^{2}+{\left(v_{Ri+}-v_{Ri-}\right)}^{2}\right)}^{1/2}}{2}\\ \sum_{k=1}^{5}a_{k}{\text{R}}_{i}^{k}=({\left({\left(x_{Ii+}-x_{Ii-}\right)}^{2}+{\left(y_{Ii+}-y_{Ii-}\right)}^{2}\right)}^{1/2}-{\left({\left(u_{Ri+}-u_{Ri-}\right)}^{2}+{\left(v_{Ri+}-v_{Ri-}\right)}^{2}\right)}^{1/2})/2 \end{array}$$
(4) When the principal point, focal length and distortion coefficients are determined, the inclination angle of the image plane can be obtained using the geometric relationship, and the average value can be calculated using the multiple-set of symmetrical points.

(18) $$|\theta_{i}|=\left|\arccos(\frac{\left|OP_{Ii}\right|}{|OP_{Ri}|+\Delta_{i}})-\beta_{i}\right|=\left|\arccos\left(\frac{{\left({x_{Ii}}^{2}+{y_{Ii}}^{2}\right)}^{1/2}}{{\left({\left(u_{Ri}-u_{0}\right)}^{2}+{\left(v_{Ri}-v_{0}\right)}^{2}\right)}^{1/2}+\Delta_{i}}\cdot \cos(\beta_{i})\right)-\beta_{i}\right|$$

Equation (18) is suitable for the symmetric points. When |*OP_Ri_*| + Δ*_i_* > |*OP_Ii_*|, *θ_i_* is defined positive; when |*OP_Ri_*| + Δ*_i_* < |*OP_Ii_*|, *θ_i_* is defined negative.

So far, the principal point, the focal length, the radial distortion coefficients and the inclination angle of image plane in one direction are obtained. The principal point, focal length, and radial distortion coefficients are suitable for the entire plane. Inclination angle in the other measurement direction can be obtained in the same manner.

##### Calibration Model Applied to the Entire Image Plane

The inclination angle in the two measurement directions is not sufficient. The ultimate goal of calibration experiment is to obtain an ideal image position of incident rays from any direction in the FOV. Based on the parameters obtained above, there are many methods to solve this problem. We adopt a coordinate transformation method, and establish a coordinate transformation framework for incident rays. Therefore, we make the following analysis.

We can obtain the position of point *P_Ii_*_+_ as (*x_Ii_*_+_, *y_Ii_*_+_, 0) in the coordinate system *CS_OXY_* by considering the longitude and latitude of the incident ray, external parameter *φ*, focal length *f* and Equations (9)–(11). Then, the straight line *O_C_P_Ii_*_+_ can be expressed as:

(19) $$\frac{x_{Ti+}}{x_{Ii+}}=\frac{y_{Ti+}}{y_{Ii+}}=\frac{k_{Ti+}-f}{-f}$$

Considering the angle ∠*P_Ii_*_+_*OP_Ti_*_+_ = |*θ_i_*| and the positive or negative of the angle value, the position of point *P_Ti_*_+_ in coordinate system *CS_OXY_* can be solved though the following equations:

(20) $$\{\begin{array}{c} \frac{x_{Ti+}}{x_{Ii+}}=\frac{y_{Ti+}}{y_{Ii+}}=\frac{k_{Ti+}-f}{-f}\\ \left(x_{Ti+},y_{Ti+},k_{Ti+})\cdot (x_{Ii+},y_{Ii+},0)=\left|\left(x_{Ti+},y_{Ti+},k_{Ti+}\right)\right|\left|\left(x_{Ii+},y_{Ii+},0\right)\right|\cos(\theta_{i}\right) \end{array}$$

Thus, the position of point *P_Ti_*_+_ is obtained (*x_Ti_*_+_, *y_Ti_*_+_, *k_Ti_*_+_). The position of point *P_Ti_*_−_ can also be obtained in the same way. So far, the positions of the multiple sets of points as *P_Ti_*_+_ and *P_Ti_*_−_ in both the two measurement direction in *CS_OXY_* can be obtained. Therefore, the plane equation of actual image plane with inclination *O′U′V′* in coordinate system *CS_OXY_* can be expressed as *p*(*O′U′V′*).

The transformation matrix from coordinate system *CS_OXY_* to coordinate system *CS_O′U′V′_* can be obtained at the same time:

(21) $$CS_{O\prime U\prime V\prime }=CS_{\text{OXY}}R_{1}(-\varepsilon )R_{2}(-\eta )$$

*R*_1_ and *R*_2_ are the coordinate system transformation matrices.

$$R_{1}(-\varepsilon )=\left(\begin{array}{ccc} 1 & 0 & 0\\ 0 & \cos(-\varepsilon ) & \sin(-\varepsilon )\\ 0 & -\sin(-\varepsilon ) & \cos(-\varepsilon ) \end{array}\right),R_{2}(-\eta )=\left(\begin{array}{ccc} \cos(-\eta ) & 0 & -\sin(-\eta )\\ 0 & 1 & 0\\ \sin(-\eta ) & 0 & \cos(-\eta ) \end{array}\right)$$

Thus, the whole parameter estimation model of the optical system of the star tracker is completed.

For any point on the image plane of the incident ray, we can determine its position in *CS_O′U′V′_* as (*u_Ri_*_+_−*u*_0_, *v_Ri+_*−*v_0_*, 0). The position of point *P_Ti_*_+_ can be calculated by considering the distortion as (*u_Ti_*_+_−*u*_0_, *v_Ti_*_+_−*v*_0_, 0). According to the coordinate system transformation matrix, the position of point *P_Ti_*_+_ in *CS_OXY_* can be calculated as:

(22) $$(x_{Ti+},y_{Ti+},k_{Ti+})\prime =R_{1}(-\varepsilon )R_{2}(-\eta )(u_{Ti+}-u_{0},v_{Ti+}-v_{0},0)\prime$$

The equation for straight line *O_C_P_Ti_*_+_ can be obtained. Further, by solving the position of the point of intersection of straight line *O_C_P_Ti_*_+_ and plane *OXY*, the point of intersection *G_Ii_*_+_ is the ideal position of point *P_Ri_*_+_. Thus, the ideal image position of the incident ray can be obtained. This solution is particularly important for the star pattern recognition and attitude solution of the star tracker.

### 4.3. Calibration Results and Discussions

#### 4.3.1. Error Analysis

**Figure 20.**
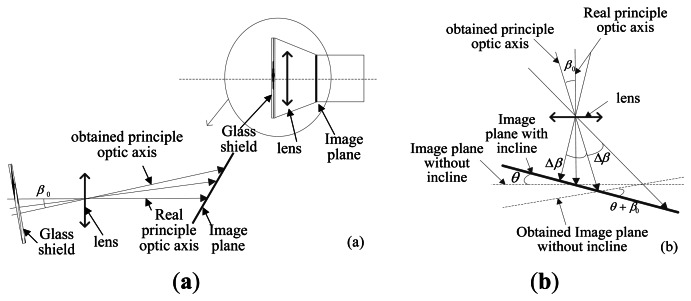
(**a**) Principal optic axis deviation error and its effect; (**b**) Its effect on the calibration process.

In the calibration process described in Section 4.2.4.2, the principal point position is obtained and considered as its true position. In reality however, due to lens installation, accuracy in manufacture as well as the limitations in the eyepiece alignment when using the theodolite, a deviation error of the optical axis is inevitable. That is to say, there may be an error *β_0_* between the optical axis obtained in Section 4.2.4.2 and the true optical axis of the lens. Maximum value of *β_0_* is approximately 40″ in this calibration system, consisting of 30″ installation error of the lens and 10″ eyepiece alignment error. In this situation, the optical axis error will cause the deviation error of principal point, the focal length and inclination angle. The specific effect analysis is described as follows:

As shown in Figure 20(b), assuming the deviation error of the optical axis is 40″ as analyzed above, the position error of principal point is about *f*·tan(40″) (equal to 0.64 pixels); the error of inclination angle is approximately 0.01°. The errors due to the focal length and the distortion are merged and the residual error of the distortion after calibration is within 0.1 pixels. Because the light ray is brighter than a real star in practical use, the signal to noise ratio is higher and the extraction error of the light ray position can reach approximately 0.05 pixels. We can use the MC error analysis method described in Section 3.3.1 to determine the *ξ_A_* and *ξ_P_*.

The *ξ_A_* meets distribution rule: *μ* = 0.0034, *σ* = 2.1192″. The *ξ_P_* meets distribution rule: *μ*= 0.0007, *σ* = 0.0338 pixels. We adopt *ξ_P_* as the basis of the calibration target. That means the calibration residual error should in the range of *μ*+3*σ* distribution of *ξ_P_*.

#### 4.3.2. Calibration Results and Discussion

The calibration experiment is conducted in the laboratory. From the above analysis, we can know the longitude angle *α_i_* and latitude angle *δ_i_* of the incident ray of the theodolite. We also can obtain the position of point as *P_Ri_*_+_ or *P_Ri_*_−_ by the image processing mentioned in Section 4.2.3. Then we do the same procedure as Section 4.2.4.3, and calculate the position of point *G_Ii_*_+_. The position of point *P_Ii_*_+_ can be also obtained through Equations (9)–(11). Comparing the two positions, the calibration model is proved to be effective and adequate if the error between the two positions is less than the *ξ_P_* for points on the image plane at any incident light angle. In the experiment, we conduct two orthogonal direction measurements to get the parameters and the calibration model. Then we conducted measurements in another two independent and orthogonal directions that have an angle of 45° or 135° with the 1st measurement direction *L*_1_ to prove the analysis and calibration model in this paper.

**Figure 21.**
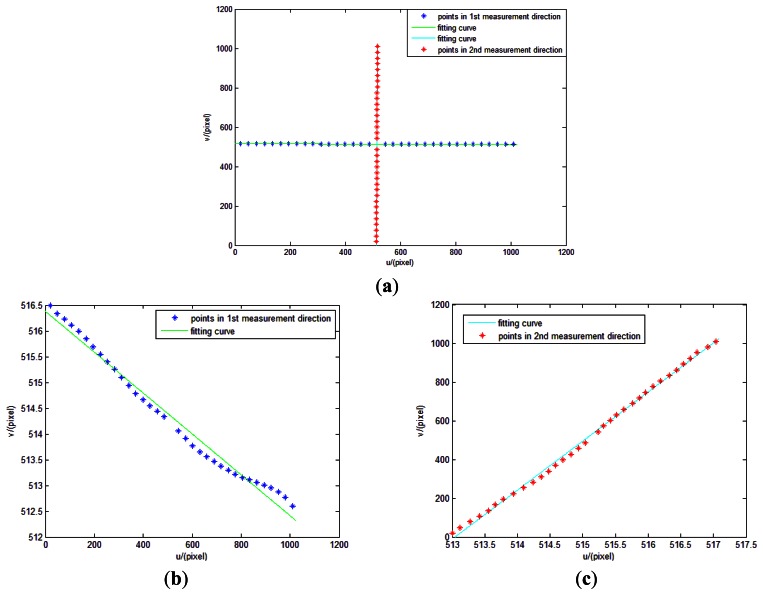
Measurement points and linear fitting curve (**a**). (**b**) and (**c**) show the two fitting curves separately.

**Figure 22.**
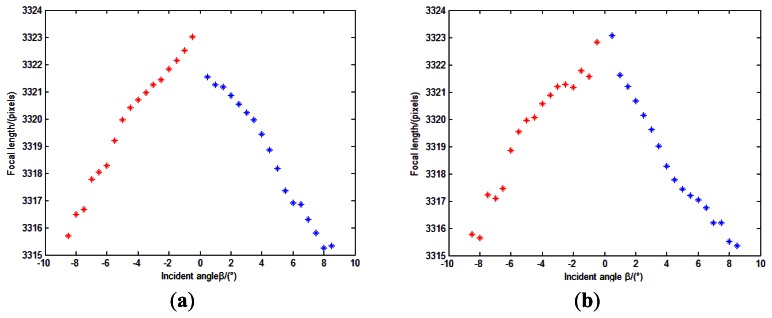
Estimations of *f*. (**a**) is the result of 1st measurement direction *L*_1_, and (**b**) is the result of 2nd measurement direction *L*_2_. Points in red and in blue represent centrosymmetry incident light rays separately.

**Figure 23.**
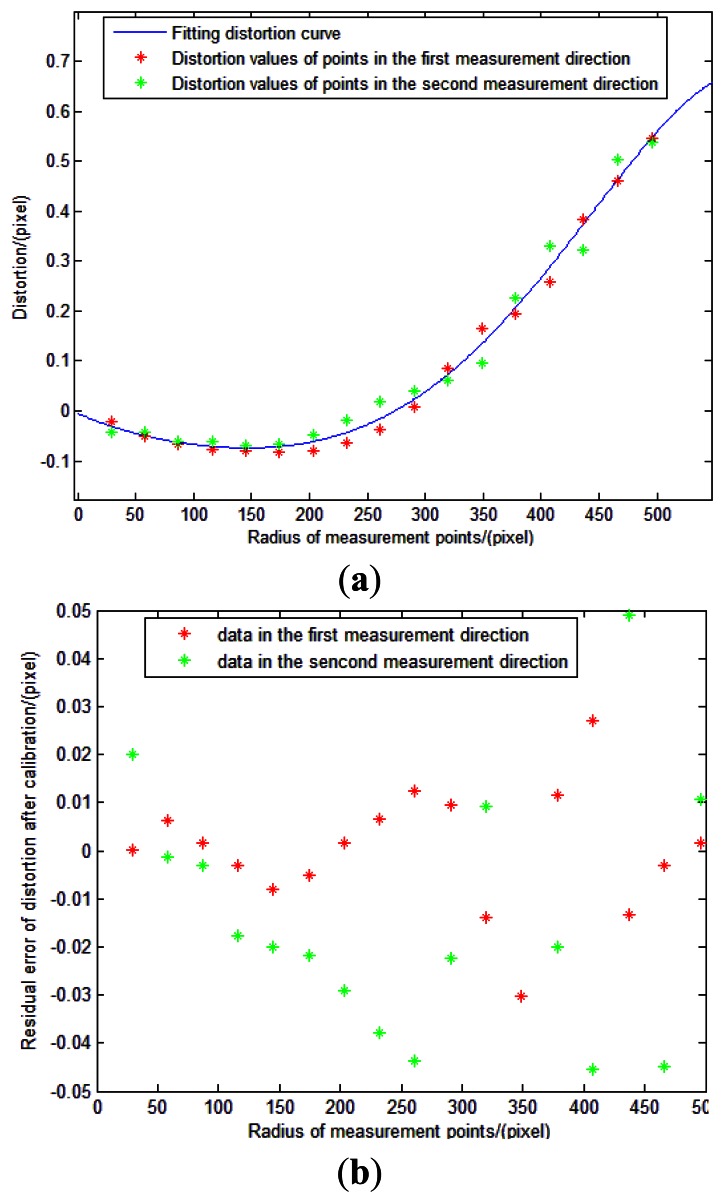
Distortion curve. (**a**) is fitting distortion curve and (**b**) is residual errors of distortion after calibration.

(1) As described in Section 4.2.4.1, we can firstly obtain external parameter *φ* using a linear fitting method. Measurement points and linear fitting curve are showed in Figure 21.
  Solving the slope of the line can determine the value of *φ*. In our experiment, the slope of the two lines are −0.003968 and 0.003949, and root mean squared errors (RMSE) are 0.1614 and 0.0576 pixels. So the values of *φ* are 0.2273° and 0.2263°. We adopt 0.2268° as the value of *φ*.
(2) As described in Section 4.2.4.2—(1), we can obtain the position of the principal point as (515.1859, 514.2069) pixels.
(3) As described in Section 4.2.4.2—(2), we can obtain a series of focal lengths utilizing the incident light in different directions and their image point (*u_Ri_*, *v_Ri_*). The estimations of *f* is shown in Figure 22.
  Theoretically, the values of the calculated focal lengths are equal. However, distortion can cause departure of the calculated focal lengths in different incident angles. Inclination of the image plane may cause deviation even when points are in centrosymmetry incident angles. Therefore, the estimations of *f* in Figure 22 is reasonable. We determine the focal length of the system as 3319.15 pixels using Equation (14). An initial estimate of *f* is adequate for calibration model as long as the radial distortion and other calibration parameters are matched with it.
(4) As described in Section 4.2.4.2—(3), we can obtain the distortion values Δ*_i_* using Equation (16). The values are shown in Table 2.
(5) We use linear least squares fitting to obtain the distortion curve. The fitting result is shown as follows, and Figure 23(b) shows that the residual error of the distortion after calibration can meet the calibration requirement analyzed in Section 3.3.

**Figure 24.**
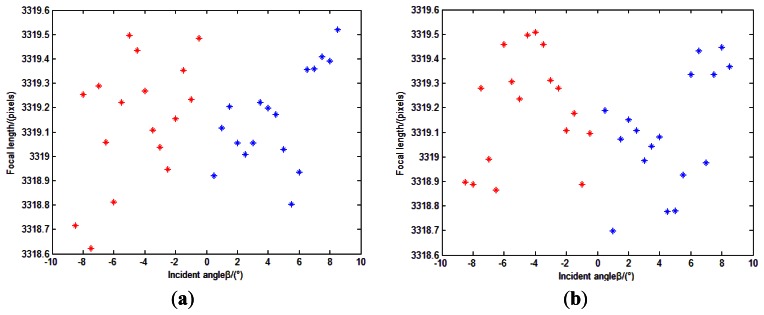
Re-estimations of *f*. (**a**) is the result of 1st measurement direction *L*_1_; and (**b**) is the result of 2nd measurement direction *L*_2_. Points in red and in blue represent centrosymmetry incident light rays separately.

**Figure 25.**
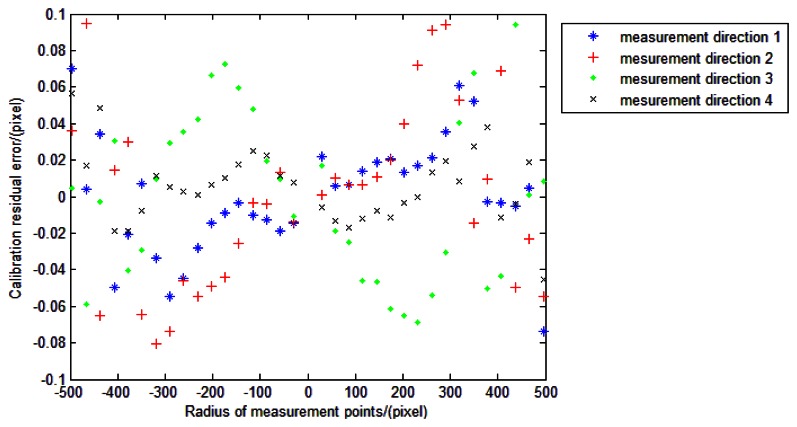
Calibration residual error.

Calibration result is summarized in Table 3:

After calibration, we can obtain the calibrated image point position of the incident light in different directions. The estimations of *f* can be conducted again to show the effect of the calibration (Figure 24) compared to the initial values in Figure 22. We can see from Figure 24 that the values of focal length do not change with the incident angles any more. They are in a range of 1 pixels (standard deviation ≈0.23 pixels). This can also demonstrate that the residual errors are improved after calibration. The position error between points *G_Ii_*_+_ and *P_Ii_*_+_ is shown in Figure 25.

We can see from Figure 25 that the position errors in the entire FOV are within the *ξ_P_* and in accordance with analysis in Section 4.3.1. The results demonstrate that the point position errors can be kept within the *ξ_P_* after calibration. Based on MC analysis, the accuracy of this calibration method can reach 7″, which related to the focal length and the FOV of the system. The accuracy of the star tracker is supposed to be better than 4.5″ after calibration, compared with the worse accuracy of more than 10″ if no calibration is conducted.

## 5. Summary and Conclusions

A synthetic error analysis approach for the star tracker has been proposed in detail in this paper. This approach can provide the error propagation relationship of the star tracker. Based on the analysis results, a calibration experiment is designed and conducted. Excellent calibration results are achieved. The calibration experiment can not only guarantee the accuracy to meet the design requirement, but can even improve the accuracy of the star tracker to a higher level. To summarize, the error analysis approach and the calibration method are proved to be adequate and precise, and are very important for the design, manufacture, and measurement of high-accuracy star trackers.

## Figures and Tables

**Table 1.** Single-error factor analysis using MC stochastic simulation.

**Item**	**Distribution of the Item**	**Distribution of*ξ****_A_*
Error of star point extraction:	μ=0, σ=0.1/3 pixels (Gaussian distribution)	μ = 0.0073, σ = 2.0281(*″*)
Error of principal point:	μ = 0, σ = 4.5/3 pixels (Gaussian distribution)	μ = −0.0139, σ = 2.0400(*″*)
Error of focal length:	μ = 0, σ = 0.6/3 pixels (Gaussian distribution)	μ = −0.0079, σ = 1.8182(*″*)
Error of inclination angle:	μ = 0, σ = 0.075°/3 (Gaussian distribution)	μ = 0.0097, σ = 1.9703(*″*)
Distortion:	μ = 0, σ = 0.1/3 pixels (Gaussian distribution)	μ = −0.0185, σ = 2.0325(*″*)

**Table 2.** Distortion values.

**Item**	**1st Measurement Direction**		**2nd Measurement Direction**	
	
	***R_i_***	Δ*_i_*	***R_i_***	Δ*_i_*
1	29.0039	−0.021951	28.9778	−0.041757
2	58.0162	−0.050355	57.9644	−0.042748
3	87.0237	−0.065911	86.9740	−0.061112
4	116.0016	−0.076456	115.9721	−0.061893
5	145.0044	−0.079762	144.9961	−0.067778
6	174.0553	−0.082395	174.0393	−0.065835
7	203.1132	−0.079229	203.0897	−0.048521
8	232.1929	−0.063527	232.1630	−0.019044
9	261.2868	−0.037760	261.2486	0.018263
10	290.4139	0.007676	290.4194	0.039629
11	319.5688	0.084626	319.6433	0.061659
12	348.7565	0.164076	348.8699	0.095181
13	378.0469	0.194652	378.0526	0.226332
14	407.3608	0.258506	407.3291	0.330992
15	436.6681	0.383765	436.7842	0.321828
16	466.1053	0.461704	466.1020	0.503454
17	495.6124	0.545419	495.6529	0.536663

**Table 3.** Calibration result.

**Item**	**Value**
*φ*(°)	0.2268
Principal point (pixels)	(515.1859, 514.2069)
Focal length *f* (pixels)	3319.15
*a*_1_	−7.038e − 04
*a*_2_	−2.344e − 06
*a*_3_	2.4705e − 08
*a*_4_	−2.7031e − 11
*a*_5_	3.1804e − 15
Inclination angle of the image plane(°) (*L*_1_ direction)	0.1469 (σ = 0.0413)
Inclination angle of the image plane(°) (*L*_2_ direction)	0.0524 (σ = 0.0221)
